# A Cross-Sectional Comparative Study of Retinal Findings in Diabetic and Non-diabetic Chronic Kidney Disease Patients

**DOI:** 10.7759/cureus.93010

**Published:** 2025-09-23

**Authors:** Sanjeet R Gandhi, Raghavendra Ijeri, Sandeep Patil

**Affiliations:** 1 Department of Ophthalmology, BLDE (DU) Shri B. M. Patil Medical College, Hospital and Research Centre, Vijayapura, IND; 2 Department of Nephrology, BLDE (DU) Shri B. M. Patil Medical College, Hospital and Research Centre, Vijayapura, IND

**Keywords:** chronic kidney disease, diabetes, dialysis and retinal changes, optical coherence tomography, retinal thickness

## Abstract

Background and objectives: Chronic kidney disease (CKD) is associated with systemic microvascular and macrovascular complications, including significant effects on the retina. While the link between diabetic retinopathy and diabetic CKD is well established, retinal involvement in non-diabetic CKD is less well recognized. Comparative evidence in the Indian population is limited, despite the high prevalence of both diabetes and CKD. This study aimed to compare retinal findings between patients with diabetic and non-diabetic CKD and to evaluate the spectrum of retinal changes across different CKD etiologies.

Methodology: This cross-sectional comparative study was conducted over 18 months at BLDE (Deemed to be University), Shri B. M. Patil Medical College, Hospital and Research Centre, Vijayapura, Karnataka, India, and included 76 patients, with 38 diabetic CKD and 38 non-diabetic CKD cases. Participants were recruited from inpatient and outpatient services and underwent detailed systemic and ophthalmologic evaluation, including visual acuity testing, fundus examination, and spectral-domain optical coherence tomography for macular and choroidal thickness. Data were analyzed using IBM SPSS Statistics for Windows, Version 20 (Released 2011; IBM Corp., Armonk, New York, United States), applying the chi-square test, t-test, and Mann-Whitney U test as appropriate, with p<0.05 considered significant.

Results: The mean age of diabetic CKD patients was significantly higher (63.52 ± 14.43 years) than that of non-diabetic CKD patients (49.26 ± 15.22 years; p < 0.001). Diabetic CKD patients also had higher serum creatinine levels (4.33 ± 1.76 vs. 3.35 ± 1.56 mg/dL; p = 0.0008) and lower eGFR (18.95 ± 3.47 vs. 22.31 ± 2.78 mL/min/1.73 m²; p < 0.001). Retinal thickness across all quadrants was significantly reduced in diabetic CKD patients (e.g., subfoveal thickness: 220.11 ± 39.67 μm in the right eye vs. 265.50 ± 28.34 μm; p < 0.001).

Conclusion: This study highlights the substantial impact of diabetes on retinal health in CKD patients. Individuals with diabetic CKD exhibited significantly more severe retinal microvascular and structural abnormalities, such as marked macular thinning, advanced stages of diabetic retinopathy, and a higher incidence of dialysis, and associated retinal changes, compared to their non-diabetic counterparts.

## Introduction

Chronic kidney disease (CKD) is an increasingly significant public health concern in India and worldwide [1], characterized by rising prevalence and substantial implications for patient health and healthcare systems [2]. According to the Global Burden of Disease Study (2017), the incidence of CKD has increased by over 29.3% since 1990, mainly due to the growing burden of diabetes, hypertension, and an aging population [2]. CKD is frequently associated with microvascular and macrovascular complications affecting multiple organ systems, including the eyes [3]. However, ocular manifestations, particularly retinal changes, often remain underrecognized in clinical practice, where systemic comorbidities tend to receive greater attention [3,4].

Diabetic retinopathy (DR), a well-established microvascular complication of long-standing diabetes, commonly coexists with diabetic kidney disease (DKD) [5]. Nonetheless, non-diabetic CKD can also lead to retinal abnormalities, as both the kidney and retina are vulnerable to similar vascular and inflammatory insults [6]. These two organs share developmental and structural similarities [6] and are subject to standard pathological mechanisms, including endothelial dysfunction, microangiopathy, oxidative stress, and chronic inflammation [7]. This underpins the “common soil hypothesis,” which proposes that systemic conditions such as diabetes and hypertension affect both organs through shared pathophysiological pathways [7,8]. As such, retinal examination is a valuable non-invasive marker of systemic vascular health, especially in CKD [7]. While the association between DR and DKD is well documented, retinal involvement is not limited to diabetes alone [8].

Numerous clinical and population-based studies in India have investigated the relationship between microalbuminuria, DKD, and DR [9-12]. Both Indian and global research have consistently demonstrated a strong correlation between albuminuria and DR in patients with type 1 and type 2 diabetes [3,10]. Retinal changes may also be observed in non-diabetic CKD, particularly in the presence of hypertension, uremia, and vascular calcification [13]. These findings underscore the importance of retinal screening in all CKD patients, irrespective of diabetic status.

Despite this, the existing literature lacks comprehensive comparative studies on retinal abnormalities in diabetic versus non-diabetic CKD patients, especially in India, where both diabetes and CKD are highly prevalent [10-14]. Most prior studies either do not differentiate between diabetic and non-diabetic etiologies or focus exclusively on diabetic populations, leaving the full range of retinal changes in non-diabetic CKD underexplored [2-4]. Moreover, the non-diabetic CKD group encompasses a heterogeneous spectrum of conditions, such as hypertensive nephrosclerosis and glomerulonephritis, contributing to variability in retinal involvement that remains insufficiently explored [12-14]. This gap hampers the development of tailored screening protocols and limits our understanding of how systemic vascular damage is reflected in retinal pathology across different CKD subgroups.

Focusing on the Indian population, this study compares retinal findings in diabetic and non-diabetic CKD patients. Specifically, it seeks to evaluate whether diabetic status influences the pattern and severity of retinal involvement, while also documenting the spectrum of retinal abnormalities across different non-diabetic CKD etiologies. By addressing these aspects, the study intends to generate evidence that can guide individualized screening strategies and provide a clearer understanding of the systemic-ocular interface of CKD.

## Materials and methods

Study setting

This hospital-based, cross-sectional comparative study was conducted over 18 months through collaboration among the Departments of Medicine, Nephrology, and Ophthalmology at BLDE (Deemed to be University), Shri B.M. Patil Medical College, Hospital and Research Centre, Vijayapura. This study involved recruiting patients with diabetic and non-diabetic chronic kidney disease from both inpatient and outpatient services for comprehensive ophthalmologic and systemic evaluation.

Definition and classification of CKD

CKD was defined according to KDIGO guidelines as abnormalities of kidney structure or function present for more than three months. Staging was based on estimated glomerular filtration rate (eGFR), calculated using the Cockcroft-Gault formula [15]. Patients were categorized as follows: Stage 1-2 (eGFR ≥60 mL/min/1.73 m²), Stage 3 (eGFR 30-59), Stage 4 (eGFR 15-29), and Stage 5 (eGFR <15). This classification was applied for diabetic (D-CKD) and non-diabetic (ND-CKD) groups, and subgroup comparisons were performed across stages to capture disease severity.

Sample size determination

Sample size estimation was based on previously published data [5], where the mean ± standard deviation (SD) of central subfield macular thickness (CSMT) in D-CKD patients was reported as 278.95 ± 45.02 µm, compared to 231.89 ± 26.72 µm in ND-CKD patients. Since optical coherence tomography (OCT)-derived retinal thickness was one of the primary outcome measures, these published values provided the most clinically relevant effect size for accurate sample size determination. Using these values, a minimum of 38 participants per group was calculated to ensure 99% statistical power at a two-sided 5% significance level. The formula applied was:

\[ N = \left( \frac{(Z_{\alpha} + Z_{\beta}) \times S}{d} \right)^2 \]

Where Zα represents the z-score corresponding to a 5% significance level (1.96), Zβ denotes the z-score corresponding to 80% power (0.84), S is the pooled standard deviation, and d is the minimum clinically relevant difference in means.

Inclusion and exclusion criteria

Participants were included if they had a confirmed diagnosis of either diabetic or non-diabetic CKD and received care from the medicine/nephrology, ophthalmology OPD, or IPD. Exclusion criteria included high myopia or developmental ocular anomalies associated with increased axial length, media opacities such as corneal scarring, cataract, or vitreous hemorrhage impeding fundus visualization, retinal or choroidal detachment, prior treatment for any form of retinopathy, or inability to undergo ophthalmic evaluation due to being bedridden. Eligible patients fulfilling the criteria were enrolled using a consecutive sampling technique, ensuring that all qualifying cases during the study period were included until the sample size was met. Written informed consent was obtained after explaining the study objectives, procedures, risks, and benefits. Participant confidentiality and voluntary participation were maintained throughout the study.

Clinical and ophthalmologic assessment

All enrolled patients underwent a structured clinical evaluation, which included recording demographic details, occupation, and thorough medical, surgical, and ocular histories. Systemic examination was performed to confirm eligibility and assess overall health status. Ophthalmological assessment involved measurement of visual acuity using a logMAR chart, indirect ophthalmoscopy to evaluate the peripheral retina, and fundus photography following pupillary dilation. Spectral-domain optical coherence tomography (SD-OCT) was performed using the ZEISS CIRRUS 500 HD-OCT system (Carl Zeiss Meditec AG, Jena, Germany), without dilation, and enhanced depth imaging (EDI-OCT) was used to measure macular and choroidal thickness. Parameters assessed included central subfield thickness (CST) and retinal thickness in subfoveal, nasal, temporal, superior, and inferior quadrants.

Nephrological and diabetic evaluation

Renal function was evaluated through serum creatinine measurements and the eGFR using the Cockcroft-Gault formula [15], which accounts for age, weight, gender, and serum creatinine levels. A correction factor of 0.85 was applied for female participants to account for differences in muscle mass. CKD stage classification (Stage 1-5) was then assigned based on the calculated eGFR described above. Glycemic control was assessed using fasting blood sugar (FBS), postprandial blood sugar (PPBS), and glycosylated hemoglobin (HbA1c), which served as an indicator of long-term glucose control.

Data analysis

All data were entered into Microsoft Excel and analyzed using IBM SPSS Statistics for Windows, Version 20 (Released 2011; IBM Corp., Armonk, New York, United States). Categorical variables were summarized as frequencies and percentages, while continuous variables were expressed as mean ± standard deviation. Appropriate statistical tests were used based on data distribution. The chi-square test was applied for categorical variables, the Mann-Whitney U test for non-normally distributed continuous variables, and the independent samples t-test for normally distributed data. All statistical analyses were two-tailed, and a p-value < 0.05 was considered statistically significant.

Comparative analysis

For comparison, participants were divided into two groups: D-CKD and ND-CKD. Further subgroup analysis was carried out according to CKD stages (Stage 1-5) to assess the influence of declining renal function on retinal and OCT changes. Variables assessed included age, gender distribution, duration of CKD, glycemic parameters, and retinal structural characteristics such as macular and choroidal thickness in nasal, subfoveal, temporal, superior, and inferior regions. This comprehensive approach enabled a detailed and clinically meaningful comparison of both ocular and systemic features between the two groups.

## Results

The mean age of diabetic CKD patients was significantly higher (63.52 ± 14.43 years) compared to non-diabetic CKD patients (49.26 ± 15.22 years; p < 0.001). Additionally, diabetic CKD patients had higher serum creatinine levels (4.33 ± 1.76 mg/dL vs. 3.35 ± 1.56 mg/dL; p = 0.0008) and lower eGFR levels (18.95 ± 3.47 mL/min/1.73 m² vs. 22.31 ± 2.78 mL/min/1.73 m²; p < 0.001) (Table 1). Among the non-diabetic CKD patients, hypertensive nephropathy was the most common etiology, observed in 12 (32%) patients. Chronic glomerulonephritis accounted for eight (21%) patients, followed by obstructive uropathy in five (13%) patients. Polycystic kidney disease (PKD) and post-infectious nephropathy were noted in four (10%) and three (8%) patients, respectively. In six (16%) patients, the underlying cause remained unknown or unspecified.

**Table 1:** Comparison of clinical and biochemical features in diabetic and non-diabetic patients with chronic kidney disease * shows a significant p-value p-value less than 0.05 was considered significant HbA1c (%): glycated hemoglobin percentage; FBS (mg/dL): fasting blood sugar (milligrams per deciliter); PPBS (mg/dL): postprandial blood sugar (milligrams per deciliter); RBS (mg/dL): random blood sugar (milligrams per deciliter); eGFR (mL/min/1.73 m²): estimated glomerular filtration rate (milliliters per minute per 1.73 square meters); CKD: chronic kidney disease

Parameter	Diabetic CKD N =38	Non-diabetic CKD N= 38	Independent t-test: Chi-square	Effect Size	p-value
Mean age (years)	63.52 ± 14.43	49.26 ± 15.22	4.01	0.97	<0.001*
HbA1c (%)	7.85 ± 1.02	5.65 ± 0.66	11.79	2.43	<0.001*
FBS (mg/dL)	141.39 ± 30.12	97.50 ± 11.55	8.36	1.74	<0.001*
PPBS (mg/dL)	216.71 ± 49.73	120.03 ± 12.36	10.92	2.34	<0.001*
RBS (mg/dL)	229.26 ± 56.32	119.84 ± 17.42	11.15	2.37	<0.001*
eGFR (mommL/min/1.73 m²)	18.95 ± 3.47	22.31 ± 2.78	-4.57	1.05	<0.001*
Serum creatinine (mg/dL)	4.33 ± 1.76	3.35 ± 1.56	2.71	0.59	0.0008*
Dialysis status			3.88, df: 1	0.24	0.049*
On dialysis	18 (47.4%)	10 (26.3%)			
Not on dialysis	20 (52.86%)	28 (73.6%)			

Table 2 shows that OCT revealed significant macular thinning in diabetic CKD patients across all measured regions. In the diabetic group, subfoveal thickness was significantly lower in both eyes (right eye: 220.11 ± 39.67 μm; left eye: 221.32 ± 41.02 μm) compared to the non-diabetic group (right eye: 265.50 ± 28.34 μm; left eye: 267.00 ± 26.85 μm; p < 0.001). A similar reduction was also noted across the superior, inferior, temporal, and nasal quadrants, indicating widespread microvascular changes in diabetic nephropathy.

**Table 2:** Comparison of retinal thickness measurements in patients with and without diabetes * shows a significant p-value CKD: Chronic kidney disease

Parameter	Diabetic CKD (Mean ± SD)	Non-diabetic CKD (Mean ± SD)	Independent t-test value	p-value
Subfoveal thickness				
Right eye	220.11 ± 39.67 μm	265.50 ± 28.34 μm	-5.87	<0.001*
Left eye	221.32 ± 41.02 μm	267.00 ± 26.85 μm	-6.16	<0.001*
Superior macular thickness				
Right eye	224.47 ± 36.77 μm	270.79 ± 31.41 μm	-5.92	<0.001*
Left eye	223.21 ± 35.26 μm	270.24 ± 29.45 μm	-6.46	<0.001*
Inferior macular thickness				
Right eye	223.58 ± 34.29 μm	268.18 ± 30.23 μm	-6.06	<0.001*
Left eye	222.63 ± 36.24 μm	268.21 ± 28.88 μm	-6.16	<0.001*
Temporal macular thickness				
Right eye	226.74 ± 38.19 μm	270.92 ± 29.50 μm	-5.74	<0.001*
Left eye	225.13 ± 35.87 μm	271.50 ± 27.63 μm	-6.35	<0.001*
Nasal thickness				
Right eye	214.47 ± 42.90 μm	260.50 ± 32.24 μm	-5.64	<0.001*
Left eye	214.50 ± 44.69 μm	261.10 ± 32.92 μm	-5.70	<0.001*

In the diabetic CKD group, normal fundus findings were noted in eight (21%) patients. Mild non-proliferative diabetic retinopathy (NPDR) was seen in nine (24%) patients, moderate NPDR in six (16%) patients, severe NPDR in 10 (26%) patients, and proliferative diabetic retinopathy (PDR) in five (13%) patients. In contrast, among the non-diabetic CKD group, normal fundus was observed in 20 (53%) patients. However, 14 (37%) patients showed grade I hypertensive retinopathy, and nine (24%) patients had grade II hypertensive retinopathy. No hypertensive retinopathy was observed in the diabetic group (Table 3).

**Table 3:** Comparison of retinal findings in chronic kidney disease patients with and without diabetes NPDR: Non-proliferative diabetic retinopathy; PDR: proliferative diabetic retinopathy; CKD: chronic kidney disease

Finding	Diabetic CKD (n=38)	Non-diabetic CKD (n=38)
Normal fundus	8 (21%)	20 (53%)
Mild NPDR	9 (24%)	0 (0%)
Moderate NPDR	6 (16%)	0 (0%)
Severe NPDR	10 (26%)	0 (0%)
PDR	5 (13%)	0 (0%)
Grade I hypertensive retinopathy	0 (0%)	14 (36.84%)
Grade II hypertensive retinopathy	0 (0%)	9 (24%)

Table 4 summarizes that patients with advanced CKD (stage 5, eGFR <15 mL/min/1.73 m²) had the most severe retinal changes. OCT findings included marked macular thinning and irregularities in the retinal pigment epithelium (RPE). Fundus examination in this group often showed severe NPDR, PDR, and optic disc pallor, indicating advanced microvascular complications. Patients in stage 4 CKD (eGFR 15-29 mL/min/1.73 m²) commonly exhibited subfoveal thinning and reduced outer retinal reflectivity on OCT. Retinal changes in this group included moderate NPDR, AV narrowing, and retinal hemorrhages. In stage 3 CKD (eGFR 30-59 mL/min/1.73 m²), OCT showed mild thinning and irregular foveal contour, corresponding to findings of mild NPDR and grade I-II hypertensive retinopathy. Patients in stages 1-2 (eGFR ≥60 mL/min/1.73 m²) generally had normal OCT findings or mild macular thinning. Fundus examination showed a normal appearance or early AV changes, indicating minimal retinal involvement in early CKD.

**Table 4:** eGFR vs retinal/OCT finding summary NPDR: Non-proliferative diabetic retinopathy; PDR: proliferative diabetic retinopathy; CKD: chronic kidney disease; OCT: optical coherence tomography

eGFR category	Number of patients	Common OCT Findings	Retinal Findings Observed
<15 (Stage 5)	8	Marked macular thinning, RPE disruption	Severe NPDR, PDR, disc pallor
15–29 (Stage 4)	22	Subfoveal thinning, reduced reflectivity of the outer retina	Moderate NPDR, AV narrowing, retinal hemorrhages
30–59 (Stage 3)	30	Mild thinning, irregular foveal contour	Mild NPDR, Grade I–II hypertensive retinopathy
≥60 (Stage 1–2)	15	Normal architecture or mild subclinical thinning	Mostly normal fundus or early AV changes

Among the 20 patients undergoing dialysis, OCT findings frequently showed changes in the RPE, irregular foveal contour, and macular thinning. These patients were more likely to have severe NPDR, optic disc pallor, and PDR, indicating more advanced retinal involvement. In contrast, the 56 patients not on dialysis mostly had near-normal OCT findings, with only mild foveal changes. Fundus findings in this group were generally less severe, with a higher proportion of mild NPDR and grade I hypertensive retinopathy. These results suggest that dialysis is associated with more pronounced retinal and OCT abnormalities, likely reflecting more advanced systemic vascular disease (Table 5).

**Table 5:** Dialysis status vs retinal/OCT changes OCT: Optical coherence tomography; RPE: retinal pigment epithelium; NPDR: non-proliferative diabetic retinopathy; PDR: proliferative diabetic retinopathy

Diabetic Status	Number of Patients	Common OCT Findings	Retinal Findings Observed
On dialysis	20	Macular thinning, irregular contour, RPE changes	Higher prevalence of Severe NPDR, PDR, disc pallor
Not on dialysis	56	Near-normal OCT, mild fovea contour alteration	More cases of Mild NPDR, Grade I hypertensive changes

## Discussion

CKD is a systemic illness associated with progressive nephron loss and widespread microvascular involvement, particularly affecting the retina [2,3]. The coexistence of diabetes mellitus further exacerbates microvascular injury, leading to more severe ocular involvement [7]. This study aimed to compare retinal and OCT findings in diabetic and non-diabetic CKD patients and to correlate these changes with eGFR and dialysis status.

In the current study, patients with diabetic CKD demonstrated significantly more frequent and advanced retinal and OCT abnormalities compared to those with non-diabetic CKD. Macular thinning, irregular foveal contour, retinal pigment epithelium (RPE) disruption, and reduced central macular thickness (CMT) were more prominent in diabetic patients, especially those in advanced CKD stages or undergoing dialysis. These findings are consistent with those of Paterson et al. [16], who reported a higher prevalence of PDR and macular thinning in stage 5 CKD. Similarly, Liu et al. [17] and Da Silva et al. [18] observed retinal microvascular abnormalities and increased arteriolar constriction in advanced CKD, underscoring the role of renal dysfunction in retinal microangiopathy.

In stage 4 CKD (eGFR 15-29 mL/min/1.73 m²), we noted subfoveal thinning, reduced reflectivity of outer retinal layers, and features of moderate NPDR. Arteriovenous (AV) narrowing, retinal hemorrhages, and hard exudates were also commonly observed. These findings align with those of Da Silva et al. [18], who reported increased AV caliber changes in patients with eGFR <30 mL/min/1.73 m². Liu et al. [17] similarly demonstrated that moderate CKD independently contributes to retinal microvascular pathology, even in non-diabetic individuals.

In stage 3 CKD (eGFR 30-59), mild foveal thinning and subtle foveal contour irregularities were the predominant OCT findings, accompanied by early NPDR or hypertensive retinopathy. Vujosevic et al. [19] reported comparable changes, including early neurodegenerative alterations and reduced central macular thickness (CMT) in patients without overt diabetic retinopathy. Chow et al. [20] also identified early thinning of the retinal nerve fiber layer (RNFL) in stage 3 CKD, highlighting the utility of OCT in detecting subclinical pathology.

Patients with eGFR ≥60 (stages 1-2) mostly showed normal OCT morphology or minor changes, such as mild parafoveal thinning. Clinically, these patients had either no retinopathy or grade I hypertensive changes. In the SEED study, Chew et al. [21] reported a low prevalence of retinopathy in early CKD, particularly among non-diabetics. Bilici et al. [22] emphasized that structural changes in early CKD may only be evident through OCT angiography or functional assessments, even when fundus examination appears normal.

Our study also identified a relationship between glycemic control and retinal abnormalities. The mean HbA1c among diabetic participants was 7.85%, indicating suboptimal control. Wong et al. [23] found that HbA1c >7.5% is associated with worsening diabetic retinopathy and structural retinal changes, while Kwak et al. [24] showed that reduced eGFR independently predicted retinopathy severity. 

Consistent with Da Silva et al. [18], our OCT-based analysis revealed that diabetic CKD patients had lower CST and subfoveal choroidal thickness than non-diabetics. Liu et al. [17] linked decreased CST with proteinuria and systemic microvascular injury. In our cohort, nasal macular thinning was most pronounced, aligning with findings by Chow et al. [20], who noted preferential involvement of the nasal and temporal macular quadrants in CKD.

Fundoscopic examination in diabetic patients revealed varying stages of DR: 24% had mild NPDR, 26% severe NPDR, and 13% PDR. In contrast, ND-CKD patients mainly exhibited hypertensive changes, with 37% showing grade I and 24% grade II retinopathy. These findings corroborate those of Chew et al. [21] SEED study, which reported more AV changes in non-diabetics and a higher prevalence of DR in people with diabetes.

Dialysis status significantly influenced both OCT and fundus findings. On OCT, patients on dialysis exhibited macular thinning, disrupted foveal architecture, and RPE abnormalities. Fundoscopy revealed higher rates of severe NPDR, PDR, and optic disc pallor. Daniel et al. [25] reported RNFL thinning in long-term diabetic dialysis patients, while Vujosevic et al. [19] observed loss of outer retinal layers and photoreceptor damage in diabetic patients on dialysis.

In contrast, non-dialyzed patients showed essentially normal or mildly abnormal OCT findings and only early NPDR or grade I-II hypertensive retinopathy. Zhang et al. [26] concluded that significant retinal microvascular and neurodegenerative changes often emerge after the initiation of dialysis, especially in poorly controlled diabetics. Dialysis-induced hemodynamic shifts and oxidative stress may further aggravate retinal ischemia.

Among non-diabetic CKD patients, hypertensive nephropathy was the most common cause, followed by chronic glomerulonephritis, obstructive uropathy, PKD, and post-infectious glomerulonephritis-similar to the distribution in the SEEK-India cohort [21]. 

Retinal changes among non-diabetics varied by underlying etiology. Hypertensive nephropathy was associated with AV narrowing and increased arteriolar reflexes, as reported by Wong et al. [23]. Patients with glomerulonephritis exhibited subtle macular thinning, potentially linked to systemic inflammation. For patients with PKD, thinning in the temporal macular quadrant was noted despite the absence of DR. These results are consistent with the study by Wan et al. [27] in which they also reported similar sectoral thinning in PKD patients. Our findings also revealed that obstructive uropathy was associated with irregular foveal contour, possibly due to chronic uremia and impaired perfusion.

Vascular caliber analysis showed that diabetic patients had larger arteriolar and venular diameters. Dumitrescu et al. [28] reported 96-120 µm arteriolar calibers and 120.5-137 µm venular calibers in diabetic individuals, consistent with our data. This may reflect endothelial dysfunction and poor glycemic control. Similar trends were noted by Wong et al. [23], who proposed retinal vessel caliber as a marker of CKD severity and DR progression.

Interestingly, several diabetic patients in our study showed subfoveal and inner retinal thinning despite no clinically detectable DR. This may represent early neuroretinal degeneration preceding vascular changes. Dumitrescu et al. [28] also noted similar OCT patterns in people with diabetes without retinopathy.

Limitations

Despite the insights provided, our study has limitations. The modest sample size may limit generalizability. Additionally, specific advanced imaging modalities such as OCT angiography, choroidal vascularity index, and electrophysiological testing were not included. These tools could have yielded a deeper understanding of early neuroretinal degeneration. Another limitation is the significant age difference between the diabetic and non-diabetic CKD groups, which may have influenced both eGFR values and retinal thickness, introducing potential bias into the results.

Furthermore, formal normality testing (e.g., Shapiro-Wilk test) was not explicitly performed before applying statistical analyses, which could affect the interpretation of some comparisons. As this was not a randomized controlled trial, all diabetic patients were not on the same antidiabetic regimen, and variations in medications could have influenced both systemic and retinal outcomes, thereby introducing treatment-related bias. In addition, potential confounders such as hypertension, duration of diabetes, and differences in systemic comorbidities were not fully controlled for, which may have influenced both renal function and retinal findings. When interpreting the results, these unmeasured variables and other sources of bias must be acknowledged. Nonetheless, the study highlights the significant effects of diabetes, declining eGFR, and dialysis on retinal health and underscores the importance of regular ophthalmic evaluation in CKD patients. Future longitudinal studies using multimodal imaging, age-matched cohorts, standardized medication regimens, and adjustment for major confounders are recommended to validate these findings further.

## Conclusions

This comparative study demonstrates the significant impact of diabetes on the retinal health of patients with CKD. Patients with diabetic CKD exhibited more severe retinal microvascular and structural abnormalities, including pronounced macular thinning, advanced stages of diabetic retinopathy, and a higher prevalence of dialysis-associated retinal changes, compared to their non-diabetic counterparts. The observed correlations between declining renal function and worsening retinal findings highlight the shared pathophysiological mechanisms affecting systemic and ocular microvasculature, such as chronic hyperglycemia, oxidative stress, and hypertension. These findings support the inclusion of routine retinal evaluations in CKD management, particularly for diabetic patients, to enable early detection of systemic vascular injury and inform timely, multidisciplinary intervention.
